# Phase 1/2 study of topical submicron particle paclitaxel for cutaneous metastases of breast cancer

**DOI:** 10.1007/s10549-022-06584-6

**Published:** 2022-04-26

**Authors:** Mario E. Lacouture, Shari B. Goldfarb, Alina Markova, Sant P. Chawla, Karan Dewnani, Marc Iacobucci, Julie E. Lang

**Affiliations:** 1 Memorial Sloan Kettering Cancer Center, New York, NY 10065 USA; 2 Sarcoma Oncology Center, Santa Monica, CA 90403 USA; 3 NanOlogy, LLC, Fort Worth, TX 76107 USA; 4 University of Southern California Keck School of Medicine, Los Angeles, CA 90033 USA; 5 Dermatology Service Department of Medicine, Memorial Sloan-Kettering Cancer Center, 530 East 74th Street, New York, NY 10021 USA

**Keywords:** Cutaneous metastases, Breast cancer, Paclitaxel, SOR007, Submicron particle paclitaxel, Clinical trial

## Abstract

**Purpose:**

This Phase 1/2 study evaluated safety and efficacy of a topical submicron particle paclitaxel (SPP) in an anhydrous ointment base (SOR007), primarily in breast cancer patients with cutaneous metastases (CM).

**Methods:**

One of three concentrations of SOR007 SPP (0.15%, 1.0%, or 2.0%) was applied twice daily over an area of 50 cm^2^ under a 3 + 3 phase 1 design for up to 28 days, with the option for expansion to an additional 28 days at the highest dose under a Phase 2a once safety was established. Efficacy was analyzed by lesion measurements and photographs to determine overall response rate (ORR), complete response (CR), and progression free survival by day 28 or 56.

**Results:**

Twenty-three subjects were enrolled, 21 with cutaneous metastases of breast cancer (CMOBC). Four subjects received SOR007 0.15% for a median of 28 days (range = 17–29), three at a dose of 1.0% for a median of 28 days (range = 6–29), and sixteen at 2.0% for a median of  55 days (range = 6–60). All doses were well tolerated, and 19 subjects were evaluable for efficacy. At day 28 across all dose levels, 16% (95% CI 3.4 to 39.6%) of subjects achieved an ORR and another 63% (95% CI 34.9–96.8%) had stable disease (SD). The proportion of patients being progression free at 28 days across all treatments was 79% (95 CI 54–94%).

**Conclusion:**

Application of SOR007 0.15%, 1.0%, and 2.0% to CM was safe and well tolerated with some reduction in lesion pain, and minimal systemic absorption of paclitaxel. Lesion stabilization was observed in most subjects over the study period. A randomized, placebo-controlled trial to confirm these findings is warranted.

**ClinicalTrials.gov identifier:**

NCT03101358.

## Introduction

CM in patients with metastatic breast cancer are not uncommon, with the prevalence being between 5 and 10% [1–4]. CM frequently present as rapidly growing and initially painless dermal or subcutaneous nodule(s) with intact overlying epidermis that can lead to ulceration, pain, discharge, malodor, bleeding, infection, and disfigurement [5, 6]. The lesions tend to grow in three-dimensions becoming raised over time. The impact on patient quality of life (QOL) can be devastating [3]. CM are also a negative prognostic factor, with a reported median time to progression in patients with untreated lesions to be less than 1 month [7].

In rare cases, CM may be the first indication of an internal malignancy but are more often a late manifestation of widely disseminated disease, foreshadowing a poor prognosis with 75% of patients dying within 12 months of first occurrence [4, 8, 9]. CM are not direct extensions of the primary cancer but form because of tumor cell transport through the lymphatics or vasculature [10]. CM are classified relative to their location: local or distant. Although many primary cancers can metastasize to the skin, the most common are breast cancer, followed by lung and gastrointestinal carcinomas [4, 11].

There are no topical drug therapies approved by the US Food and Drug Administration (FDA) for the treatment of CM. This, in conjunction with paclitaxel’s broad-spectrum cytotoxic mechanism-of-action, provided the basis for the investigation of submicron particle paclitaxel (SPP), suspended in an anhydrous base (SOR007), as a topical treatment for CM. The potential advantages of topical interventions over systemic therapy include ease of administration, decreased distress, reduced toxicity and the creation of a feeling of patient-directed control [8]. Based on preclinical studies, it was anticipated that SPP would facilitate the transport of paclitaxel through the skin into the dermis, providing a continuous tumoricidal dose of paclitaxel to CM over the dosing interval with limited systemic exposure. It was also anticipated based on previous preclinical and clinical studies that the application of SOR007 SPP to CM and the surrounding skin would be well tolerated, allowing for repeat self-administration to large areas of the body if necessary.

## Materials and methods

In this open label, single arm Phase 1/2a dose escalation and expansion clinical trial, 23 subjects with non-melanoma CM, the underlying metastatic cancer confirmed prior to consent by preferred institutional methodology, which included biopsy, conventional radiography, and/or imaging (CT, MRI, PET, etc.), were treated with one of three concentrations of topical SOR007 (0.15%, 1.0%, or 2.0%), applied twice daily for up to 28 or 56 days. Each topical application was one fingertip unit containing 0.75 mg, 5 mg, or 10 mg of SPP over a 50 cm^2^ treatment area via a 3 + 3 dose design for up to 28 days, with the option for additional 28 days of treatment at the highest dose once the maximum tolerated dose (MTD) was determined. Subjects were screened and eligibility was confirmed up to 14 days prior to enrollment. Eligible subjects had an ECOG Grade 0–2 with a minimum life expectancy of 3 months. Subjects were ineligible with open or ulcerated wounds extending through the dermis within the treatment area. The study included a dose escalation and dose expansion phase.

Lesion response was determined by the percent change relative to baseline in the sum of the longest diameter of all eligible lesions (using RECIST 1.1 criteria) at end of treatment recorded on day 29 or 57. Overall Response Rate (ORR) was defined as the proportion of those subjects with complete response (CR) or partial response (PR). Progression Free Survival (PFS) was defined as the proportion of those subjects with CR or PR or stable disease (SD) at end of treatment. Best overall response (BOR) was defined as the best measured response recorded from day 15 to end of treatment. Time to treatment failure (TTF) was defined from the start of therapy to treatment failure for any reason, including progressive disease, treatment discontinuation, treatment toxicity, or death, lesion-specific pain was assessed at study visits using a Numeric Rating Scale (NRS-11) [12]. Biopsies were not included in the analysis of lesion response to treatment because of the associated significant morbidity given the history of radiation and frequent bacterial colonization of CMOBC [5].

The dose escalation phase followed a 3 + 3 dose-ascending design, where the first cohort received 0.15% SOR007, the second 1.0% SOR007, and the third 2.0% SOR007 twice daily for up to 28 days. Dose escalation was based on approval by the Safety Monitoring Committee (SMC). The SMC reviewed all available data after the last subject in each cohort of three subjects who completed 15 days of SOR007 SPP applications to determine whether dose escalation could be continued up to the top dose of 2.0%.

In the dose expansion phase of the current study, additional subjects were enrolled at the dose level determined to be the maximum tolerated dose (MTD). In this phase, a decision was made by the investigator to continue treatment for up to 56 days, or discontinue sooner, based on the subject’s disease status, systemic therapy requirements, treatment tolerability, and subject consent.

Subjects began twice-daily dosing on Day 1 and continued up to Day 28 during the dose-escalation and up to Day 56 in the dose-expansion phase of the trial. Study visits were scheduled at Days 8, 15, 29, and 43 for subjects treated for 28 days, and at Days 8, 15, 29, 57 and 70 for subjects treated for 56 days. All subjects had a safety assessment 30 days after the last SOR007 SPP application.

### SOR007 SPP treatment area selection and lesion measurement

At baseline (Day 1), the Investigator identified and outlined with a marker a 50 cm^2^ area containing at least one eligible lesion (per RECIST criteria version 1.1, lesions ≥ 10 mm diameter in the longest diameter). The Investigator confirmed the primary eligible lesion measurement by caliper. If multiple lesions were present in the treatment area the Investigator had the option to measure and track additional lesions during the trial. New lesions appearing in the 50 cm^2^ area were also measured and tracked until healed or until study end. At baseline (Day 1) and at each study visit, the longest diameter and perpendicular width were measured, and photographic documentation of the treatment area was taken using Image J JAVA image processing.

### Pharmacokinetic analysis (PK)

Blood was drawn 24 h after the first application of ointment, and at each study visit. The PK sample collection window was 24-h post Day 1 (± 2 h), prior to the first daily application on Days 8 and 15, and any time during Days 29, 43, 57, and 70.

### Lesion pain

The Numeric Rating Scale (NRS-11) was used to assess lesion pain [11–13]. The scale was anchored by two verbal pain severity descriptors, one at each symptom extreme: “no pain” (score of 0) and “worst imaginable pain” (score of 10). Instructions were provided to subjects to verbally describe average pain intensity (best and worst) in the previous 24 h and current intensity at each visit.

### Imaging

At least two global and two close-up photographs were taken at each study visit using a standard focal length digital camera with the intent of analyzing lesion response via Image J (an image processing program in the public domain developed at the NIH) to confirm site measurements. Image to image analysis of change in two-dimensional surface area was used for analysis of individual lesion BOR in subjects.

### Statistical analysis

Patient characteristics and clinical outcomes data were presented as descriptive statistics as means, medians, or proportions, with 95% CI. TTF was evaluated as a time to event analysis using the method of Kaplan–Meier. There were no formal comparative analyses between dosing cohorts.

### Preparation of SOR007 SPP 0.15%, 1.0%, and 2.0%

Supercritical precipitation (SCP) technology is a process by which a drug substance dissolved in an organic solvent is precipitated in a supercritical antisolvent. Baltezor et al. [14] described a method by which paclitaxel dissolved in acetone processed with supercritical CO_2_ [15] creates SPP with a number-weighted mean particle size that can be approximately 0.8 microns, with a specific surface area greater than 18 m^2^/gm and a bulk density between 0.05 and 0.15 gm/cm^3^. SPP concentrations of 0.15%, 1.0%, and 2.0% were suspended in an anhydrous preservative free base (SOR007) consisting of compendial grades of cyclomethicone, mineral oil, paraffin wax, and white petrolatum and filled into a 15-g laminated tube and supported by 3 years of room temperature stability.

## Results

Twenty-three subjects were enrolled into the study (Table 1). Subjects had a mean age of 63 years, 21 were female diagnosed with breast cancer and two were male (one with leiomyosarcoma and one with extramammary Paget’s disease). Seventeen (74%) received prior chemotherapy and/or were receiving chemotherapy during study enrollment. Most of the sample was white (61%) and had an ECOG PS of 0 or 1 at enrollment (91%). All patients had metastatic disease at enrollment, with the most common sites of metastases being the bone (26%), lung (26%) and brain (22%). In post-trial follow up, investigators reported 14/23 (61%) had a skin biopsy prior to enrollment.

**Table 1:** Subject demographics and baseline characteristics

Parameters	Total (*N* = 23)
Age (Mean; range)	63 (38–75)
Sex	
Female	21 (91%)
Male	2 (9%)
Body Mass Index [(kg/m^2^) Mean (SD)]	26 (5)
Race	
Asian	3 (13%)
Black or African American	3 (13%)
White	14 (61%)
Other	3 (13%)
Cancer diagnosis	
Breast	21 (91%)
Other	2 (9%)
ECOG performance status	
0	8 (35%)
1	13 (56%)
2	2 (9%)
Site of metastases	
Bone	11(48%)
Lung	6 (26%)
Liver	4 (17%)
Brain	5 (22%)
Other	8 (35%)

*ECOG* Eastern cooperative oncology group

SOR007 was well tolerated at all dose levels, with only one patient experiencing grade 3 or 4 events, that were not considered to be treatment related (Table 2). No abnormal laboratory safety results (hematology, chemistry, or urinalysis) or changes in vital signs were considered related to study drug. Unexpected local skin reactions were mild to moderate in severity and not (superficial chest wound, blisters, infection) or unlikely (3 cases of bleeding) related to the study drug based on Investigator assessment. There were also no dose limiting toxicities (DLTs) identified allowing escalation to the highest dose (2.0%). Of all enrolled subjects, 4 received 0.15% SOR007, 3 received 1.0% SOR007, and 16 received 2.0% SOR007 (11 of the 16 to the 56-day treatment endpoint).

**Table 2:** Overview of treatment emergent adverse events (TEAEs)

Adverse events	All doses (*n* = 23)		
	All Grades *n* (%)	Grades 1–2 *n* (%)	Grades 3–4 *n* (%)
Skin lesion	7 (30%)	6 (26%)	1 (4%)
Application site pain	7 (30%)	7 (30%)	–
Pruritus	7 (30%)	7 (30%)	–
Application site hemorrhage	6 (26%)	5 (22%)	1 (4%)
Hyperglycemia	5 (22%)	5 (22%)	–
Headache	4 (17%)	3 (13%)	1 (4%)
Aspartate aminotransferase increased	4 (17%)	4 (17%)	–
Fatigue	3 (13%)	2 (9%)	1 (4%)
Nausea	3 (13%)	2 (9%)	1 (4%)
Cough	3 (13%)	3 (13%)	–
Blood alkaline phosphatase increased	3 (13%)	3 (13%)	–
Hypomagnesaemia	3 (13%)	3 (13%)	-

TEAEs that were observed in greater than 10% of all subjects (*n* ≥ 3) are listed in table

Adverse Event is coded using MedDRA version 20.1; Grade 1—Mild, Grade 2—Moderate, Grade 3—Severe, Grade 4—Life threatening

*TEAE* treatment emergent adverse events

Measurements of longest dimension and perpendicular width of each target lesion were taken by the investigator at each subject visit throughout the study. Lesion response was analyzed at baseline and at end of day 28 or 56 to treatment to determine ORR and PFS using the change in the sum of longest dimension of all target lesions under RECIST 1.1. The 2 subjects receiving 0.15% SOR007 were not included in the lesion response analysis because 1 withdrew prior to the day-15 measurement and the other 1 had insufficient data for analysis. One subject receiving 2.0% SOR007 withdrew from the study at day 6 and another had insufficient data for analysis. In all, 19 subjects receiving 0.15% (*n* = 2), 1.0% (*n* = 3) or 2.0% (*n* = 14) SOR007 were evaluated after 28 days of treatment and 8 of the 11 subjects receiving 2.0% SOR007 for 56 days of treatment. At the day 28 and day 56 assessment, ORR was 16% (3/19) and 25% (2/8), respectively (Table 3). Similarly, at day 28 and day 56, 15/19 (79%) and 6/8 (75%) were progression free (RECIST 1.1). BOR, defined as the best measured response recorded from day 15 to end of treatment, was 21% (Table 3). The data also suggested a dose-duration response relationship but will require a larger trial study to confirm. Median TTF could not be calculated in either the 28 day or 56-day groups because there were too few events. Indeed, in 11 evaluable subjects within the 28-day cohort, there were only two patients with documented lesion progression. In the 56-day cohort, there were only two patients with documented lesion progression of 9 evaluable subjects.

**Table 3:** Clinical outcomes data

Lesion response by subject (RECIST 1.1)							Individual lesion response (All subjects)			
	28-day (*n* = 19)		56-day (*n* = 8)		BOR (*n* = 19)		BOR (Longest Dimension) (*n* = 36)		BOR (Area) (*n* = 41)	
	Count	Percent	Count	Percent	Count	Percent	Count	Percent	Count	Percent
CR	0	0%	0	0%	0	0%	4	11%	4	10%
PR	3	16%	2	25%	4	21%	8	22%	10	24%
SD	12	63%	4	50%	13	68%	18	50%	17	41%
PD	4	21%	2	25%	2	11%	6	17%	10	24%
ORR	3	16%	2	25%	4	21%	12	33%	14	34%
PFS	15	79%	6	75%	17	89%	30	83%	31	76%

Abbreviations/Definitions: 28-DAY = 2 subjects treated with 0.15% SOR007 for 28 days, 3 subjects treated with 1.0% SOR007 for 28 days, 14 subjects treated with 2.0% SOR007 for 28 days; 56-DAY = response at 56 days for the 8 subjects treated with 2.0% SOR007 for a total of 56 days; CR = complete response meaning absence of any detectable residual disease in eligible lesion(s) in the treatment area; ORR = overall response rate (CR + partial response [PR]); Progression Free Survival (PFS) [CR + PR + stable disease (SD)]; *BOR* best overall response from day 15 measurement to end of treatment. Lesion Response by Subject sum of longest dimension of each target lesion per subject analyzed via site dimensions; Individual Lesion Response BOR of all lesions treated with SOR007 by longest dimension and by Area (measured in two dimensions via ImageJ, an image processing program in the public domain developed at the National Institutes of Health

**Fig. 1:**
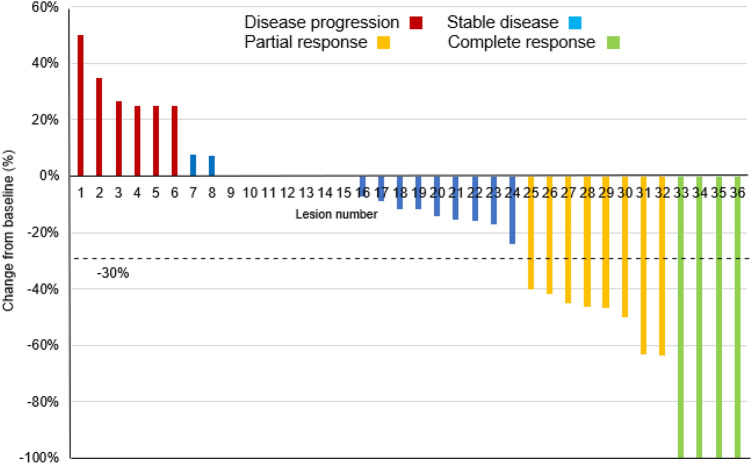
Best overall response to SOR007 in 36 cutaneous lesions from 19 patients (RECIST 1.1)

**Fig. 2:**
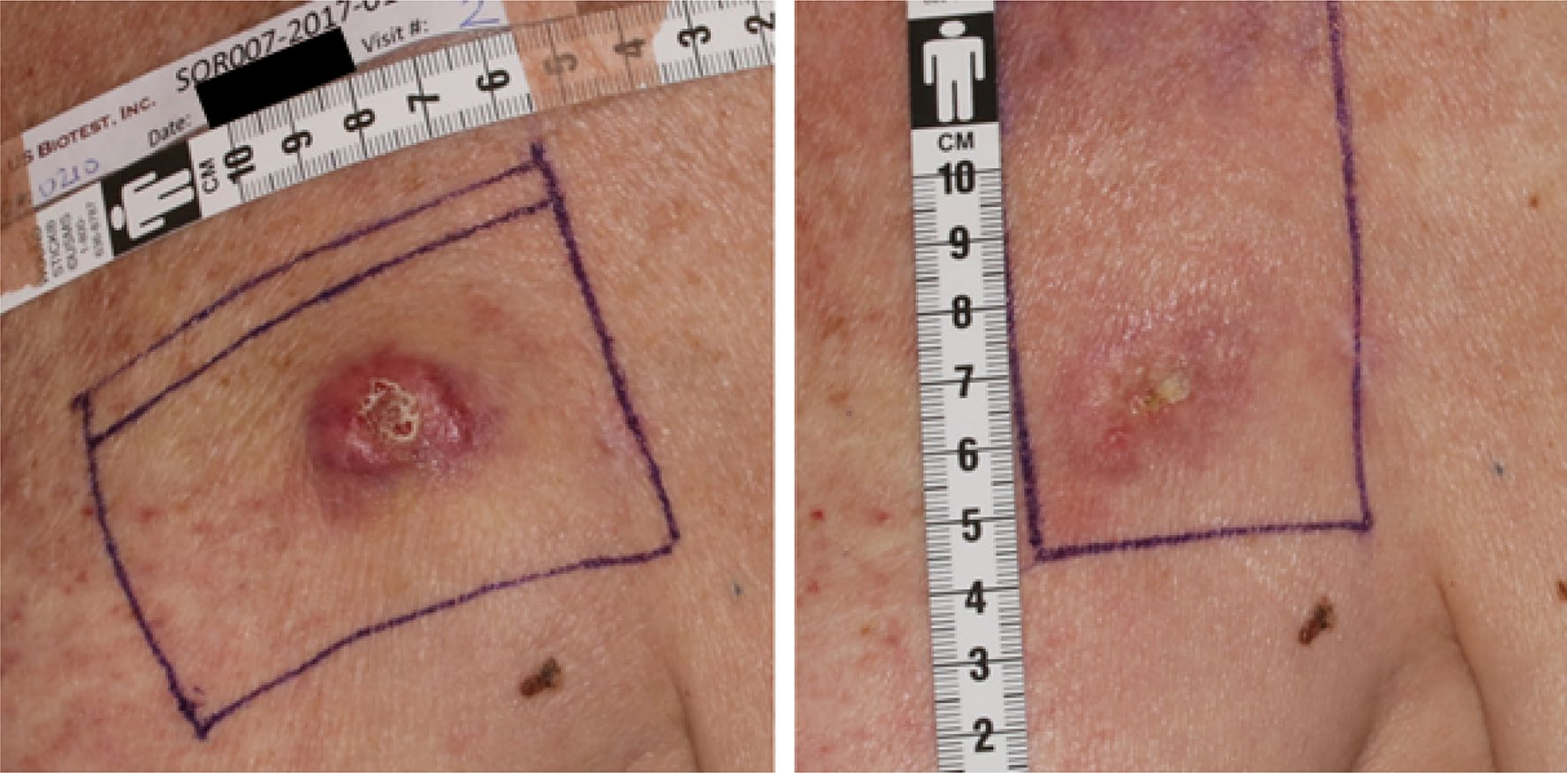
Example of CMOBC lesion prior to treatment (**a**) with SOR007 2.0% and at end of study (**b**) recorded as complete response

BOR by individual lesion for longest dimension utilized dimensions of 36 lesions identified by the sites and BOR by individual lesion for area utilized area of 41 lesions identified by Image J analysis. The BOR by individual lesion for longest dimension (*n* = 36) versus area (*n* = 41) for CR was 4/36 (11%) versus 4/41 (10%), for ORR 12/36 (33%) versus 14/41 (34%) and for PFS 30/36 (83%) versus 31/41 (76%) suggesting reasonable correlation between dimension and area analysis. A waterfall plot of the 36 individual lesions measured by dimension shows that 21/36 (58%) decreased in size at end of SOR007 treatment including 4 lesions (11%) with a complete response (Fig. 1). An illustration of a CMOBC lesion prior to treatment (a) with SOR007 2.0% and at end of study (b) recorded as complete response, is presented in Fig. 2.

### Lesion pain

At baseline, 11 of 23 patients (48%) reported lesion pain. Of these, 7 of 11 (64%) reported a decrease of at least one unit in their pain score relative to baseline, 3 (27%) reported a worsening of their pain, and 1 (9%) reported no change in pain (Table 4). In the 7 patients who reported a decrease in pain, follow up data at day 15 following the completion of treatment was also collected. Two of 7 patients reported stabilization in pain scores (i.e., no change from the end of treatment measurement), while 5 of 7 patients reported a worsening of pain (Table 4) at day 15 following the completion of treatment. The clinical relevance of a minor change in pain score (i.e., single unit reduction) was not evaluated in this study.

**Table 4:** Pain response in evaluable patients

Pain outcome	Number of subjects (*n* = 23)
Number experiencing lesion pain at enrollment	48% (11)
Change in lesion pain score at end of treatment assessment	
Decrease	64% (7)
No change	9% (1)
Increase	27% (3)
Change in lesion pain score 15 days post treatment in subjects reporting a decrease at end of treatment (*n* = 7)	
Decrease	0% (0)
No change	29% (2)
Increase	71% (5)

### Pharmacokinetics

PK evaluation was conducted in 23 subjects at multiple timepoints. Plasma paclitaxel concentrations were below the lower limit of quantitation (LLOQ of 25 pg/mL) at all timepoints in all but two subjects. In these subjects, peak plasma paclitaxel concentrations of 36 pg/mL on day 8 and 103 pg/mL on day 29 were recorded, which is less than 3% the systemic toxicity threshold for paclitaxel.

## Discussion

Despite the prevalence and QOL impact of cutaneous metastases (CM) in cancer patients, topical therapy standard-of-care (SOC) remains elusive. Treatment is complicated by underlying metastatic disease, poor patient performance status, previous radiation therapy, and short survival duration. These factors make surgery and radiation untenable for most patients [11, 16, 17]. Systemic chemotherapy also has a negligible impact on most CM [1, 18]. Additional therapies include electrochemotherapy (ECT), photodynamic therapy (PDT), intralesional therapy (ILT), and topical therapy (TT). In a meta-analysis of 47 prospective studies of 4313 CM lesions, the objective response rate to the therapies (including radiotherapy) was 60% [1]. ECT has demonstrated benefit in an uncontrolled, retrospective study (complete response [CR] rate 48%); however, ECT often requires general anesthesia and results in notable pain and dermatologic toxicity (inflammation, hyperpigmentation, and ulceration) [17]. Additionally, ECT appears to be more successful in smaller lesions (measuring less than 4 cm^2^), making it unreliable for larger lesions [19]. The less invasive alternatives, such as ILT and TT, demonstrated reduced efficacy in comparison to ECT. Therefore, a significant unmet need exists for an effective, localized, non-invasive therapy for CM in patients with advanced disease, either as monotherapy or in combination with other agents.

Paclitaxel is a highly protein-bound hydrophobic molecule, with a high molecular weight, which limits its access to tumor cells. IV Administration of paclitaxel achieves relatively low drug levels in tumor cells that are of short duration, resulting in limited cancer cell exposure. Submicron particle paclitaxel (SPP), with a number-weighted mean particle size of approximately 0.8 microns (calculated to contain approximately 2 billion paclitaxel molecules each), with a high surface area to facilitate drug release from the pure paclitaxel particles [15], was formulated in a patented anhydrous preservative-free topical base, which demonstrated paclitaxel permeability through the stratum corneum, epidermis, and into the dermis in in vitro cadaver skin studies [20].

This was the first study of SOR007 SPP administered topically twice daily for up to 56 days in cancer patients with CM. Of the 23 subjects enrolled, most (21 of 23) had CMOBC and had been previously treated with or were concurrently receiving systemic chemotherapy. Although the primary endpoint of this study was safety and tolerability, preliminary assessments of efficacy was assessed by lesion measurement changes and patient-reported pain scores. Overall, the majority of subjects showed stabilization or reduction of lesions and the response of non-eligible lesions to the study drug were consistent with those of eligible lesions. There was also limited systemic absorption of paclitaxel, with only 3 subjects having detectable levels despite a LLOQ of 25 pg/mL. These PK results are similar to a previous study of topical SOR007, which showed negligible systemic absorption of paclitaxel [21]. Given the negligible absorption of paclitaxel from SOR007, it is unlikely drug-related systemic toxicities would occur with longer term treatment.

Improvements in patient-reported lesion pain, assessed with the NRS-11 were also reported, with some patients being pain-free after SOR007 application. Considering the FDA’s emphasis on patient-focused drug development, improvement in patient-reported pain represents an important endpoint of clinical benefit. The data suggest that SOR007 SPP may have a positive impact on patient-perceived lesion pain. These improvements would not be expected based on the natural history of CM as untreated patients with CM have been reported to experience persistent symptoms and deteriorating QOL [7, 22]. SOR007 was also well-tolerated by study subjects, with a toxicity profile superior to systemic chemotherapy, radiation, and ECT. This can be attributed to the lack of serious local skin reactions, negligible systemic paclitaxel absorption found in the PK analysis, and the absence of DLTs allowing for dose-expansion to the SOR007 2.0%.

Notwithstanding these encouraging findings, there were several study limitations that need to be acknowledged. The trial had no comparator arm as it was primarily designed to evaluate safety. As such, there is no way to evaluate the significance of the observed clinical benefits of SOR007 SPP seen in this trial. There are few randomized, placebo-controlled, clinical trials of topical drug therapies in CM to provide a historical reference data on lesion response and pain reduction. One such study, however, of topical miltefosine solution in 52 patients with CMOBC reported a median time to lesion progression of 21 days in the placebo group highlighting the aggressive tendency of these lesions [7]. Keeping in mind the caveats of cross trial comparisons, a PFS of 79% after 28 and 75% at 56 days of SOR007 treatment was observed. It must also be noted, however, that 28 or 56 days may be too short to adequately evaluate lesion progression. In addition, the clinical relevance of a one unit decrease in lesion pain may be of minimal significance. Both parameters should be more extensively evaluated in the next trial. Furthermore, the sample size in the current study was small and skin biopsy to confirm CM was not captured in the clinical study report, which limits the external validity of the findings. Confirmation in a larger randomized clinical trial is needed.

## Conclusion

The findings from this early dose escalation/expansion study indicate that concentrations up to 2.0% SOR007 are safe, well tolerated and may provide clinical benefit to patients with CM not responsive to systemic agents. Overall, a majority of subjects had lesion stabilization or improvement over the 28 or 56-day study period, some lesion pain reduction during treatment, and negligible systemic paclitaxel absorption. Results from this study will inform additional trials of topical administration of SOR007 SPP focused on CMOBC.

## Data Availability

The datasets generated during and/or analyzed during the current study are available from the corresponding author on reasonable request.
